# Hybrid approach for left-sided colonic carcinoma obstruction; a case report

**DOI:** 10.1186/1477-7819-9-42

**Published:** 2011-04-21

**Authors:** Atthaphorn Trakarnsanga, Thawatchai Akaraviputh, Asada Methasate, Vitoon Chinswangwatanakul

**Affiliations:** 1 Minimally Invasive Surgery unit, Division of General Surgery, Department of Surgery, Faculty of Medicine Siriraj Hospital, Mahidol University, Bangkok, Thailand

**Keywords:** Left-sided colonic obstruction, Colonic stent, Single-incision laparoscopic colectomy

## Abstract

Traditionally, there are several approaches to manage left-sided colonic carcinoma obstruction, such as tumor resection with primary anastomosis, tumor resection with end-colostomy and loop-colostomy. Recently, colonic stent insertion was introduced as a bridge prior to definite surgery. We demonstrated a hybrid approach for obstructed sigmoid carcinoma using colonic stent, followed by single incision laparoscopic colectomy (SILC). A 58 year-old man presented with complete left-sided colonic obstruction. He underwent emergency colonoscopy with metallic stent placement. One week later, he was performed SILC. He recovered well after the operation without any postoperative complications. The pathological result showed adequacy of oncologic resection. This hybrid approach of colonic stent insertion and SILC can be safely performed.

## Background

Eight to twenty-nine percent of colorectal cancer patients presented with colonic obstruction [1-4]. The obstruction of colon is one of the most common emergency presentations of colorectal cancer, especially lesion at left-sided, which frequently causes morbidity and mortality. Management of left-sided colonic obstruction can be done in several ways such as tumor resection with primary anastomosis (one-staged), tumor resection with end-colostomy (two-staged) and emergency transverse loop-colostomy. Interestingly, recent publications supported the colonic stent insertion as a bridging therapy before definite surgery. Traditional approach, patients usually ended up with stoma. From previous reports, one-third of stomas are never reversed [1,5]. For this reason, colonic stent may prevent undesirable colostomy.

Laparoscopic colectomy for colon cancer treatment became more popular in the past decade. The data from several studies [6-13] showed better short-term benefits, such as less postoperative pain due to smaller incision, rapid return of bowel function, shorter hospitalization and faster return to normal activities. Moreover, short-term complications, morbidities and mortality were not different from traditional approach. Importantly, there are no difference of oncologic outcomes between laparoscopic and open surgery in terms of local recurrence and percentage of adequate margin [6-13]. For these reasons, laparoscopic colectomy was not inferior to conventional surgery and provided better short-term advantages [14]. Recently, there were several minimally invasive surgical techniques introduced, for examples, Hand-assisted laparoscopic colectomy (HALC), Single incision laparoscopic colectomy (SILC) as well as Robotic-assisted laparoscopic colectomy.

The approach with placement of colonic stent before laparoscopic colectomy seems to be an ideal approach for left-sided colonic obstruction [15-18]. However, there is no report that showed result of sequential procedures, comprising of an insertion of colonic stent, followed by SILC. We demonstrated our new hybrid approach for obstructed sigmoid cancer.

## Case presentation

**Figure 1:**
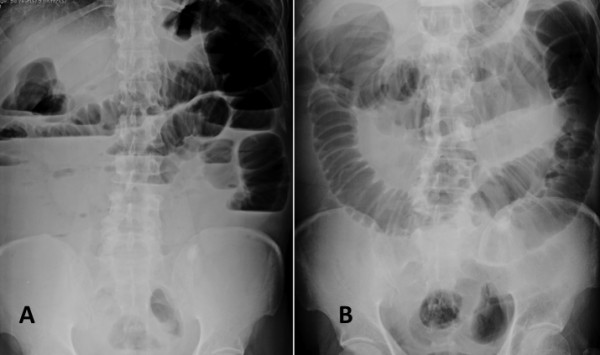
**Plain abdominal films, supine (A) & upright (B), revealed left-sided complete colonic obstruction**.

We reported a 58-years-old male (BMI = 17.3 kg/m^2^) with underlying of seizure who presented with abdominal distention, constipation and vomiting 4 days. On physical examination revealed dehydration, abdominal distention and hyperactive bowel sound. Plain abdominal films revealed dilation of small bowel and large bowel till sigmoid colon. (Figure 1) The limited barium enema was performed and showed obstruction from a circumferential mass at sigmoid colon. He underwent a new hybrid approach comprising of an insertion of colonic stent follow by SILC.

### Operative Techniques

#### Colonic stent placement

**Figure 2:**
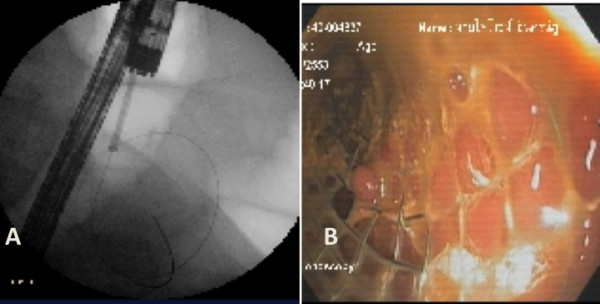
**A self-expandable metallic stent (Wallflex^® ^90 mm, Boston Scientific) was inserted over the wire technique under endoscopic (A) and fluoroscopic (B) control**.

After general anesthesia was administered and endo-tracheal tube was inserted. Patient was laid in left lateral decubitus. Therapeutic colonoscopy was used and showed circumferential ulcero-proliferative lesion at 25 cm from anal verge. Sphincterotome and guide wire was passed under fluoroscopy. Contrast was injected via sphincterotome catheter to confirm the position. Colonic stent (Wallflex ^® ^90 mm, Boston Scientific) was placed over the wire (Figure 2). After procedure, the patient went well. He could pass flatus and stool. We gave him liquid and low residual diet. We used milk of magnesia 30 ml once a day for bowel preparation. One week later, we scheduled him for SILC.

#### Single incision laparoscopic colectomy (SILC)

**Figure 3:**
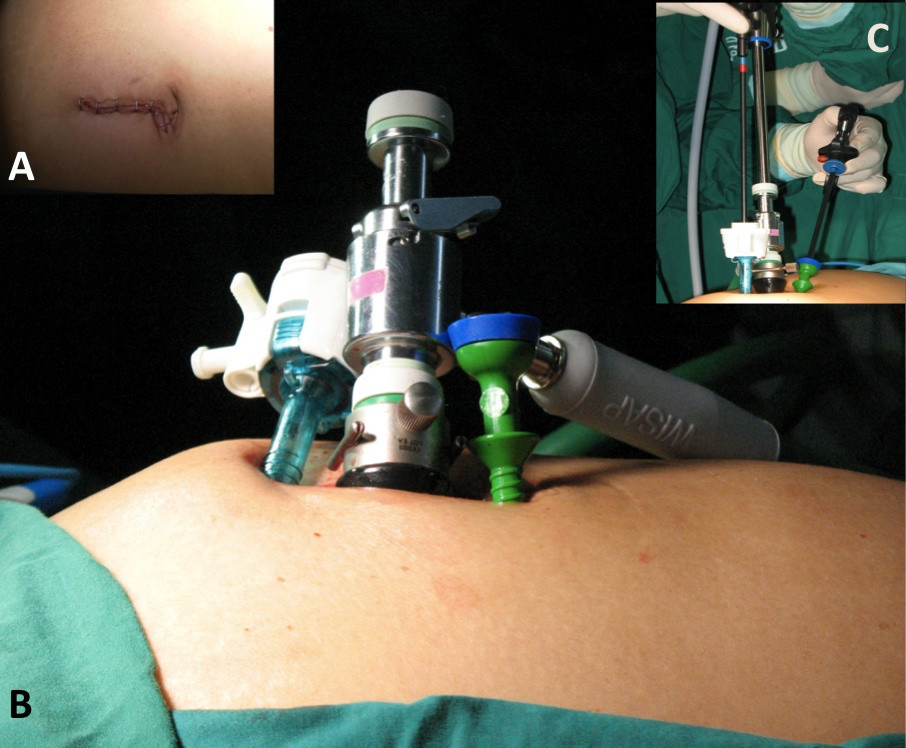
**A small sub-umbilical incision was about 5 cm in length (A)**. Hasson's trocar in the center and two of 5 mm ports were introduced with multi-fascial technique via the incision (B). **A **30-degree camera (Endoeye™, Olympus), endohook (right) and bowel grasper (left) were used for dissection (C).

After general anesthesia was administered. Patient was placed in modified Lithotomy position. Small sub-umbilical incision was made about 5 cm (Figure 3A). Skin and subcutaneous tissue was divided until anterior of abdominal sheath. Pnuemoperitoneum was created by closed technique with Veress needle until adequate pressure around 15 mmHg. Hasson's trocar was introduced to abdominal cavity. 10 mm 30 degree camera (Endoeye™, Olympus) was inserted. Two of 5 mm ports were placed at the upper and lower end of incision, respectively. (Figure 3B) Straight laparoscopic 5 mm instruments, using endohook (monopolar cautery) on the right-hand side and bowel grasper on the other side were used for dissection (Figure 3C).

**Figure 4:**
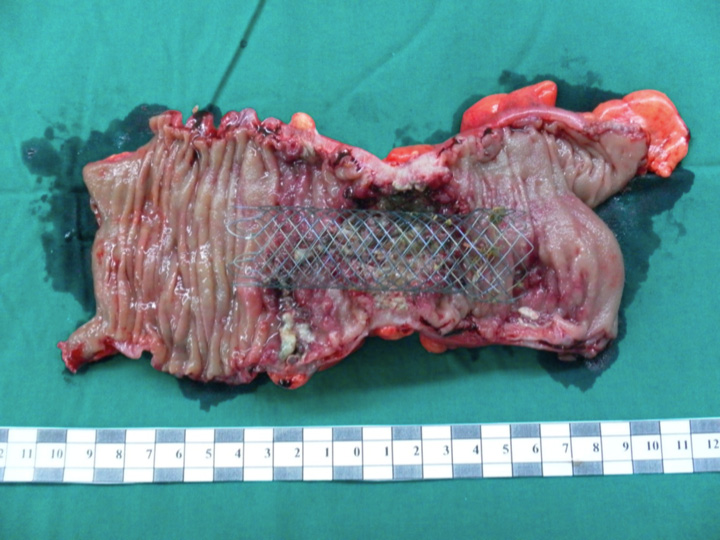
**The specimen of sigmoid colon showed constricted circumferential ulcero-proliferative lesion with the self-expandable metallic stent inside**.

Sigmoid colon was mobilized using medial to lateral approach. Left ureter was the first landmark for this step after that sigmoid artery was identified, clipped (Hem-o-lok^®^, Teleflex) and divided. The dissection was continued proximally to splenic flexure and upper rectum distally. Sheath was incised continuously. Wound retractor (Allexis^®^, Applied Medical) was applied. Left sided colon was bringing to abdominal wall. Resected specimen was removed. (Figure 4) Side to side Colo-colostomy was performed with staple anastomosis (GIA™ 80 mm and TA™ 60 mm, Covidien). The operative time was 185 min and blood loss was less than 100 ml. No blood transfusion was needed.

After the operation, he returned to ordinary ward in stable condition. He recovered very well and oral fluid could be started on day second after the operation. He could be discharged on postoperative day sixth without any complication. The pathological result confirmed adenocarcinoma, moderately differentiated, sized was 5.7 × 4.0 × 2.8 cm. Tumor was invaded to visceral peritoneum (pT4a) and presented of perineural invasion. Two of 14 lymph nodes showed metastatic carcinoma (pN1a). Proximal and distal margins were uninvolved. At present he is doing well.

## Discussion

Malignant left-sided colonic obstruction is the most common etiology for emergency condition in colon cancer [19]. Traditionally, most of the patient needed emergency surgery and some of them ended up with stoma. Stoma never reverses up to one-third of the patients [1,5]. This situation can changed quality of life of them. Recently, several techniques was used to avoid the stoma formation, for example subtotal colectomy and primary anastomosis, intraoperative colonic lavage and colonic stent insertion as a bridge to surgery that change emergency to elective surgery.

Dohmoto [20] who is the first described using colonic stent for relief of colonic obstruction in 1991. The indication for insertion of colonic stent is palliative treatment in advanced cancer and using as a bridge to surgery [21]. For bridging therapy, there are several advantages in various groups because the need of emergency surgery can be avoided up to ninety percent of cases [22,23]. More time of preoperative evaluation lead to benefit for patients with high surgical risk. In addition, open standard surgery may be avoidable in candidates for laparoscopic surgery because of more time for good bowel preparation.

There are several studies showed the good results when use colonic stent as a bridge before laparoscopic surgery [24-27]. Park et al [24] compared 25 patients in stent-laparoscopic surgical group (SLAP) and 70 patients in open surgery with intraoperative colonic lavage group (OLAV). Operative time was shorter in SLAP (198.53 vs. 262.17 min, *P *= 0.002). Oral intake was resumed earlier in SLAP (5.18 vs. 6.65 days, *P*< 0.001). Similarly of positive results, Cheung et al [25] reported a randomized controlled trial of obstructing tumor between the splenic flexure and rectosigmoid junction in adult patient. Twenty-four underwent endoluminal stenting followed by laparoscopic surgery and 24 under went open surgery. Significantly successful of one-stage procedure in endo-laparoscopic group was reported (16 vs. 9 *P *= 0.04). None of the patient in endo-laparoscopic group had a permanent stoma compared with 6 patients in the open surgery group (*P *= 0.03). However there is a recent RCT from France [28] which could not demonstrated that emergency preoperative SEMS for patients presenting with acute left-sided malignant colonic obstruction could significantly decrease the need for stoma placement. Regard to the outcome, 17 patients in the surgery group sustained a stoma placement versus 13 patients in the SEMS group (p = 0.30). In this multicenter trial, they revealed high rate of stent placement failure and stent perforation, leading to premature closure of the study before the expected number was reached.

Single incision laparoscopic surgery is a new emerging laparoscopic technique, which performed colonic resection through only one incision. Some papers proposed of safety and feasibility of this technique [15-17]. In addition, SILC seems to be improved in cosmetic with potential decreased pain and fewer incidence of postoperative incisional hernia [18,19,29,30]. However, laparoscopic manipulation of a bulky sigmoid tumor with a stent in-situ would be technically very challenging even with the conventional laparoscopic approach. One should not embark on this hybrid approach if they don't have enough experience with SILC. There still are various possible operative options for this situation. After removal of the affected bowel, the discontinuity can be treated by Hartmann's procedure, or continuity can be restored by anastomosis in an open or laparoscopic technique. This hybrid approach might be used for a thin patient with a small colorectal tumor by high experience laparoscopic colorectal surgeon.

This is the initial report for management of left-sided colonic obstruction with colonic stent insertion followed by SILC that showed the good results of recovery period. Moreover, oncologic resection is still adequate. However, we need more patients to enroll for the further study.

## Conclusions

In Summary, a new hybrid approach using colonic stent as a bridging therapy combine with SILC can be performed with promising results. This approach might be a good alternative way to manage left-sided colonic obstruction. Nevertheless, to determine its benefits, larger prospective comparative studies to standard open or laparoscopic colectomy with cost analysis, oncologic outcomes, and long-term follow-up will be necessary.

## Consent

Written informed consent was obtained from the patient for publication of this case report and any accompanying images. A copy of the written consent is available for review by the Editor-in-Chief of this journal.

## Competing interests

The authors declare that they have no competing interests.

## Authors' contributions

AT and TA designed study and performed the operation. AT and TA wrote the paper. All authors read and proved the final manuscript.
